# A randomized phase II study to assess the effect of adjuvant immunotherapy using α-GalCer-pulsed dendritic cells in the patients with completely resected stage II–IIIA non-small cell lung cancer: study protocol for a randomized controlled trial

**DOI:** 10.1186/s13063-017-2103-4

**Published:** 2017-09-15

**Authors:** Hideo Saka, Chiyoe Kitagawa, Yukito Ichinose, Mitsuhiro Takenoyama, Hidenori Ibata, Tatsuo Kato, Koji Takami, Motohiro Yamashita, Tadashi Maeda, Sadanori Takeo, Hitoshi Ueda, Kan Okabayashi, Seiji Nagashima, Tadayuki Oka, Hidenori Kouso, Seiichi Fukuyama, Kentaro Yoshimoto, Mototsugu Shimokawa, Akiko M. Saito, Suminobu Ito

**Affiliations:** 1 Department of Respiratory Medicine, National Hospital Organization Nagoya Medical Center, Aichi, Japan; 2 Department of Thoracic Oncology, National Hospital Organization Kyushu Cancer Center, Fukuoka, Japan; 3 Department of Respiratory Medicine, National Hospital Organization Mie Chuo Medical Center, Mie, Japan; 4 Department of Respiratory Medicine, National Hospital Organization Nagara Medical Center, Gifu, Japan; 5 Department of Respiratory Medicine, National Hospital Organization Osaka Medical Center, Osaka, Japan; 6 Department of Thoracic Surgery, National Hospital Organization Shikoku Cancer Center, Ehime, Japan; 7 Department of Medical Oncology, National Hospital Organization Yamaguchi Ube Medical Center, Yamaguchi, Japan; 8 Department of Thoracic Surgery, National Hospital Organization Kyushu Medical Center, Fukuoka, Japan; 9 Department of Surgery, National Hospital Organization Fukuoka Hospital, Fukuoka, Japan; 10 Department of Thoracic Surgery, National Hospital Organization Fukuoka Higashi Medical Center, Fukuoka, Japan; 11 Department of Respiratory Medicine, National Hospital Organization Nagasaki Medical Center, Nagasaki, Japan; 12 Department of Surgery, National Hospital Organization Ureshino Medical Center, Saga, Japan; 13 Department of Thoracic Surgery, National Hospital Organization Oita Medical Center, Oita, Japan; 14 Department of Thoracic Surgery, National Hospital Organization Beppu Medical Center, Oita, Japan; 15 Department of Thoracic Surgery, National Hospital Organization Minami Kyushu National Hospital, Kagoshima, Japan; 16 Clinical Research Center, National Hospital Organization Kyushu Cancer Center, Fukuoka, Japan; 17 Clinical Research Center, National Hospital Organization Nagoya Medical Center, Aichi, Japan; 18 Clinical Research Center, National Hospital Organization, Tokyo, Japan; 19 4-1-1 Sannomaru, Naka-ku, Nagoya, 460-0001 Japan

**Keywords:** Non-small cell lung cancer, Immunotherapy, Phase II

## Abstract

**Background:**

As the toxicity associated with the α-GalCer-pulsed dendritic cell (DC) therapy could be considered to be negligible, its addition to postoperative adjuvant chemotherapy would be expected to greatly improve the therapeutic effect, and could result in prolonged survival.

The aim of the present study is to compare the therapeutic efficacy of alpha-galactosylceramide-pulsed DC therapy in patients who have undergone a complete resection of stage II–IIIA non-small-cell lung cancer (NSCLC) followed by postoperative adjuvant therapy with cisplatin plus vinorelbine, to that in patients who did not receive additional treatment (surgical resection plus postoperative adjuvant chemotherapy only).

**Methods:**

Subsequent to the complete resection of NSCLC, followed by the administration of cisplatin plus vinorelbine dual-agent combination adjuvant chemotherapy, patients who satisfy the inclusion criteria will be randomly allocated to either the α-GalCer-pulsed DC immune therapy group, or the standard treatment group.

In total, 56 patients will be included in the study. The primary endpoint is recurrence-free survival, and the secondary endpoints are natural killer T-cell-specific immune response, the frequency of toxic effects and safety, and overall survival.

**Discussion:**

In order to determine the efficacy of α-GalCer-pulsed DC therapy, the present study compares patients with stage II–III NSCLC who underwent complete surgical resection followed by postoperative adjuvant therapy with cisplatin plus vinorelbine, to those who did not receive additional treatment (surgical resection plus postoperative adjuvant chemotherapy only).

**Trial registration:**

UMIN000010386 (R000012145). Registered on 1 April 2013.

UMIN-CTR is officially recognized as a registration site which satisfies ICMJE criteria.

**Electronic supplementary material:**

The online version of this article (doi:10.1186/s13063-017-2103-4) contains supplementary material, which is available to authorized users.

## Background

Primary lung cancer accounts for approximately 20% of all cancer-related deaths, with 71,518 deaths reported worldwide in 2012. Lung cancer is the primary cause of cancer-related mortality in Japan. As non-small-cell lung cancer (NSCLC) accounts for ≥80% of all lung cancers, it is imperative to manage this disease. Although surgical resection is the conventional method for the early treatment of lung cancer, patients with stage II–III disease have poor prognoses even after complete resection.

As recurrence is common after conventional surgery, powerful suppression of distant metastases and of potentially existing micrometastatic lesions throughout the body is important for the improvement of survival. Therefore, to control the micrometastatic lesions, postoperative adjuvant chemotherapy is often administered to advanced-stage patients. In order to improve the surgical performance, a treatment strategy combining chemotherapy with surgery has been attempted in three clinical trials since 2003 (The International Adjuvant Lung Trial [1], JBR.10 [2], and Adjuvant Navelbine International Trialist Association [3]). Although the efficacy of postoperative adjuvant chemotherapy was confirmed in a long-term follow-up observational study [4], the incorporation of the Adjuvant Lung Cancer Project Italy [5] and the Big Lung Trial [6] into five comparative trials resulted in a meta-analysis of the data from 4584 cases of lung cancer (Lung Adjuvant Cisplatin Evaluation (LACE) [7]). The results of the meta-analysis showed that, compared to the group that did not receive any postoperative treatment (standard treatment group), the group that received postoperative adjuvant chemotherapy had significantly longer disease-free survival (DFS; hazard ratio (HR), 0.84; 95% confidence interval (CI), 0.78–0.91; *p* < 0.001) and overall survival (OS) rates (HR, 0.89; 95% CI, 0.82–0.96; *p* = 0.005). In the present study, we showed that, compared to those in the standard treatment group, the 5-year DFS and OS rates improved by 5.8% and 5.4%, respectively, in the postoperative adjuvant chemotherapy group.

In the LACE subgroup analysis [8], patients who received cisplatin plus vinorelbine were confirmed to have superior survival rates compared to patients who received other cisplatin-based postoperative adjuvant chemotherapies. The cisplatin plus vinorelbine treatment did not improve the 5-year survival rate in stage IB NSCLC patients compared to the standard treatment group; however, for stage II and stage III NSCLC cases, the 5-year survival rates of the treatment groups improved by 11.6% (HR, 0.74; 95% CI, 0.60–.91) and 14.7% (HR, 0.66; 95% CI, 0.53–0.83), respectively. Meanwhile, toxicity grade ≥3 was observed in 90% and 49% of the patients who received combination treatments with vinorelbine and other combination treatments, respectively. The frequency of toxicity was higher in the patients treated with the vinorelbine-based combination treatments than in those treated with other combination treatments. The treatment-related death rate was 1.4%. Although cisplatin plus vinorelbine treatment showed a high frequency of toxicity, it was also efficacious in reducing the recurrence-associated mortality rate by approximately 20% compared to the standard treatment group. Therefore, cisplatin plus vinorelbine is the current standard regimen for postoperative adjuvant chemotherapy.

Natural killer T (NKT)-cells are unique cells expressing both T-cell receptors and natural killer (NK)-cell receptors on their surfaces [9]. The T-cell receptor on the NKT-cell surfaces is composed of an extremely limited α-chain (Vα24Jα18 in humans) and a β-chain (Vβ11 in humans). The ligand for this receptor is the CD1d molecule which is an antigen-presenting molecule similar to the major histocompatibility complex class-I ligand. NKT-cells recognize α-galactosylceramide (α-GalCer), a type of glycolipid displayed by CD1d, and are specifically activated to rapidly produce interferon-γ (IFN-γ) and interleukin-4 in large volumes. At the same time, they demonstrate powerful cytotoxic activity through perforin/granzyme B [10, 11]. In addition to its direct antitumor effect, the NKT-cells also regulate the damaging activity of NK-cells, CD8^+^ T-cells, and other effectors or dendritic cells (DCs). Thus, they can be considered to be unique cells [12, 13].

In a mouse model, an increase in intrapulmonary NKT-cell numbers and IFN-γ production was observed after the intravenous administration of α-GalCer-pulsed DCs [14, 15]. In a mouse lung metastasis model, it was possible to eliminate the already small, established lung metastatic tumors [16, 17].

In order to assess the powerful antitumor effects of the NKT-cells, from 2001 onward, we administered α-GalCer-pulsed DC therapy to 11 patients with unresectable advanced-stage and postoperative recurrent NSCLC at Chiba University [18]. The initial number of cells treated was 5 × 10^7^ units/m^2^. This number was increased to between 2.5 × 10^8^ and 1 × 10^9^ units/m^2^ to assess the safety of intravenously administered treatment modalities. The procedure was repeated four times. The NKT-cell-specific immune response and its antitumor effects were assessed. No toxic events of more than grade 2 were observed, showing complete fulfillment of the safety protocols. Among the patients who showed grade 2 or higher toxic events, none of the patients required treatment despite the presence of test value abnormalities such as in the form of fever, and minor increases such as in the aspartate aminotransferase (AST) level. In order to assess the NKT-cell-specific immune response, we analyzed the peripheral blood NKT-cell counts and the IFN-γ production capacity of the NKT-cells. With the administration of the maximum cell count, a clear increase was observed in the peripheral blood NKT-cell count of three patients. In addition, a further increase and reinforcement of IFN-γ production from NKT-cells was observed in one patient. Although none of the patients showed clear tumor shrinkage, one of the three patients who received the maximum cell administration volume showed disease progression; however, they achieved a survival period of 59 months with good quality of life.

Subsequently, we have performed α-GalCer-pulsed DC therapy in a phase I–II trial that commenced in March 2004 [19]. Subsequent to the administration of standard treatment, patients with inoperable advanced-stage lung cancers, or postoperative patients showing recurrence, received 1 × 10^9^ units/m^2^ of α-GalCer-pulsed DCs. As a result, in 17 of the 23 enrolled patients, the protocol could be completed. In one patient, recurrence of deep-vein thrombosis and grade 3 toxic events were observed, and hospitalization and treatment were required. Among patients with grade 2 or higher toxic events, none of the patients required treatment despite the presence of test value abnormalities in the form of fever, etc.; minor increases in AST level, etc.; and a decrease in hemoglobin levels. On analysis of the NKT-cell-specific immune response, a clear increase in the cell counts of α-GalCer-reactive IFN-γ-producing peripheral blood mononuclear cells was observed in 10 patients. Although significant changes were not identified in terms of tumor size, the median survival rate for all 23 patients was 17.4 months. Ten patients with increased IFN-producing cells (more than two-fold) showed prolonged median survival time (MST) (31.9 months; range, 14.5 to 36.3 months) as compared with poor-responder patients (*n* =7) MST (9.7 months; range, 3.8 to 25.0 months) (log-rank test, *p* = 0.0015) [19].

Upon the meta-analysis of large-scale clinical trials (LACE) for the standard cisplatin-based adjuvant chemotherapies, the postoperative adjuvant chemotherapy group showed a 5.8% improvement in the 5-year recurrence-free survival (RFS) rate compared to the standard treatment group. During subgroup analysis, the 5-year survival rates of the cisplatin plus vinorelbine treatment group were observed to have improved by 11.6% for stage II NSCLC, and by 14.7% for stage III NSCLC, compared to those of the standard treatment group. However, the treatment group also showed strong toxicity, leading to the conclusion that the therapeutic effects of cisplatin plus vinorelbine were inadequate.

As the toxicity associated with the α-GalCer-pulsed DC therapy could be considered insignificant, its addition to postoperative adjuvant chemotherapy would be expected to greatly improve the therapeutic effect, and could result in prolonged survival.

## Methods/design

### Setting and participants

This study is a multi-institutional, joint, randomized, and non-blind phase II trial with the following 15 participating institutions: Nagoya Medical Center, Kyushu Cancer Center, Nagara Medical Center, Mie Chuo Medical Center, Osaka National Hospital, Shikoku Cancer Center, Yamaguchi-Ube Medical Center, National Kyushu Medical Center, Fukuoka Hospital, Fukuoka Higashi Medical Center, National Hospital Nagasaki Medical Center, NHO Ureshino Medical Center, Oita Medical Center, Beppu Medical Center, and Minami Kyushu National Hospital.

The present study began in March 2013 and will conclude in March 2020.

### Treatment methods

**Fig. 1:**
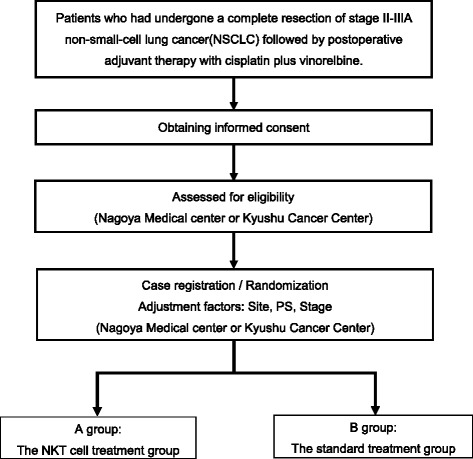
Entire design of this trial

Subsequent to the complete resection of NSCLC followed by the administration of cisplatin plus vinorelbine dual-agent combination adjuvant chemotherapy, patients who satisfy the inclusion criteria will be randomly allocated to either the α-GalCer-pulsed DC immune therapy group or the standard treatment group (multi-institutional, joint, randomized, non-blind phase II trial) (see Fig. 1).

**Fig. 2:**
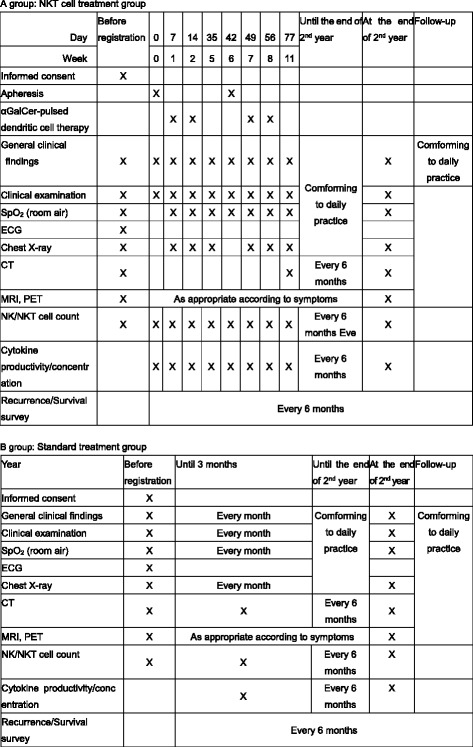
Treatment schedule

In the NKT-cell treatment group, α-GalCer-pulsed DCs formulated using the collected blood component will be intravenously administered twice weekly for one cycle, with two cycles performed in total (see Fig. 2).

The duration of each cycle is indicated below:

- Cycle 1: from the time of initial blood component collection until before the start of cycle 2, or until the termination date if treatment is terminated during the same cycle
- Cycle 2: from the second round of blood component collection until 3 weeks after second dendritic cell administration in cycle 2, or until the termination date if treatment is terminated during the same cycle

### Follow-up

The follow-up period is determined to be 2 years after the final allocation of patient enrollment.

### Sample size calculation

In the ANITA study [3], the 2-year event-free survival rate was less than 60% in all subjects who received cisplatin + vinorelbine (stage IB–stage IIIA stage; of these, stage IB 36%). When it is considered that more than 30% of subjects had disease stage IB, it can be assumed that the 2-year event-free survival rate (for stage II–stage IIIA) is approximately 50%. Moreover, when it is considered that the 2-year event-free survival rate, for subjects in stage IB who received a cisplatin-based regimen in the IALT study [1], was similar (more than 30%), the rate can be estimated to be approximately 50%. Based on these results, we calculated that the 2-year event-free survival rate of the standard treatment group should be 50%. Assuming that, in comparison with the standard treatment group (50%), 25% amelioration can be expected, the amelioration rate would be 75% in the NKT-cell treatment group. Also, when it is hypothesized that the survival function follows an exponential distribution, the HR would be 0.415. The enrollment period is 3 years and follow-up period lasted 2 years. When assuming that the significance level is set at 20% for both tails, and that the statistical power is 80%, 24 subjects per group are required (necessary event number is 23). In the event that subjects are determined to be ineligible or are excluded after enrollment, the planned number of subjects enrolled per group is 28 subjects (total of 56 subjects).

### Eligibility criteria

#### Inclusion criteria

1. Histologically confirmed NSCLC
2. Underwent hilar lymph node dissection and mediastinal lymph node dissection, lobe resection, or additional procedures including selective dissection
3. Complete resection of NSCLC (complete resection is defined as complete removal of the tumor at the time of surgery based on macroscopic examination, and the absence of tumor cells along the pathological resection line)
4. Diagnosed with stage IIA/IIB/IIIA cancer
5. Aged 20–75 years at the time of enrollment
6. Recurrence-free
7. Eastern Cooperative Oncology Group performance status of 0 or 1 at the time of enrollment
8. Undergoing treatment with cisplatin (total dose, ≥200 mg/m^2^) and vinorelbine (total dose, ≥100 mg/m^2^) dual-agent combination adjuvant chemotherapy (three to four cycles), implemented 4–16 weeks after the final administration at the time of enrollment
9. Fully maintained major organ (e.g., bone marrow, liver, and kidneys) functions that satisfy the criteria
10. Peripheral blood NKT-cell count of ≥10 units/mL
11. Full explanation of the trial details were provided; written informed consent was obtained from each patient prior to enrollment

#### Exclusion criteria

1. Presence of serious complications, including severe infectious diseases and malnutrition
2. Presence of postoperative pleural effusion, abdominal effusion, and pericardial effusion requiring treatment
3. Presence of active multiple cancers
  - Simultaneous multiple cancers and metachronous multiple cancers with disease-free periods within 5 years
  - Intraepithelial carcinoma or intramucosal carcinoma that could be cured by localized treatment
4. Ingestion or intravenous administration of corticosteroids
5. Presence of autoimmune disease
6. History of hepatitis (however, if positive for HBs and/or HBc antibodies, then HBV-DNA detection (minus) can be performed)
7. Positive for HBs antigen, HCV antibody, HIV antibody, or HTLV-1 antibody
8. Severe heart disease (New York Heart Association class III or higher) or lung disease (Hugh-Jones class III or higher)
9. History of albumin hypersensitivity
10. Pregnant, has the potential to become pregnant, or nursing
11. Blood component collection is contraindicated in case of unstable angina, AV block stage II or higher, Wolff-Parkinson-White syndrome, complete left bundle branch block, systolic blood pressure of ≤90 or ≥170 mmHg)
12. Decision of the attending physician against participation in the clinical research

### Blinding and randomization

This study is non-blind. Subjects will be randomly assigned to group A (NKT-cell treatment group) or group B (standard treatment group) at a ratio of 1:1. A dynamic minimization method using facility, Performance Status (PS), and stage (stage II versus stage III) as adjustment factors is utilized when performing random assignment to ensure that large deviations do not occur.

### Outcomes

#### Primary outcomes

1. RFS

#### Secondary outcomes

1. NKT-cell-specific immune response
2. Toxic events and safety evaluation
3. OS

### Statistical analyses

The main analysis will be performed on the full analysis set, and the per-protocol set will be included in the analysis for reference. The main objective of this trial is to compare the RFS in the NKT-cell treatment group to the standard treatment group. The null hypothesis is that the RFS period is the same in both the NKT-cell treatment group and the standard treatment group. In order to test this hypothesis, a stratified log-rank test will be performed using non-facility allocation as adjustment factors for the levels. The statistical significance is set at 20% for both groups. The Kaplan-Meier method is used for estimating the RFS curve and for calculating the annual RFS, the median RFS, and the confidence interval (CI) by NKT-cell treatment group. Based on the results of the log-rank test, the null hypothesis is disproved. In the event that the RFS curve for the NKT-cell treatment group is higher than that of the standard treatment group, NKT-cell treatment can be considered a treatment option that can further be validated in a phase III trial. For reference, the Cox proportional hazards model is used for estimating the difference in treatment effects between the two groups. In addition, a non-stratified analysis is implemented for reference.

Secondary analysis concerning the OS period is implemented for targeting the full analysis set, and analysis targeting the per-protocol set is implemented for reference. A stratified log-rank test is performed using non-facility allocation as the adjustment factor for the levels for group comparisons. For reference, all *p* values < 0.05 are considered statistically significant in both groups. The Kaplan-Meier method is used to estimate the OS curve, and we subsequently calculate the OS rate, the median OS period value, and the CI by treatment group. The Cox proportional hazards model is used to estimate the difference in treatment effects between the two groups. For reference, a non-stratified analysis is implemented.

### Interim analysis

No interim analysis will be performed during the course of the study.

## Discussion

In order to determine the efficacy of α-GalCer-pulsed DC therapy, the present study compares patients with stage II–III NSCLC who underwent complete surgical resection followed by postoperative adjuvant therapy with cisplatin plus vinorelbine, to those who did not receive postoperative adjuvant chemotherapy Additional file 1.

## Additional file

Additional file 1: SPIRIT 2013 Checklist: recommended items to address in a clinical trial protocol and related documents*. (DOC 123 kb)

## Abbreviations

AST: Aspartate aminotransferase
CI: Confidence interval
DCs: Dendritic cells
DFS: Disease-free survival
HR: Hazard ratio
IFN-γ: Interferon-γ
LACE: Lung Adjuvant Cisplatin Evaluation
NK: Natural killer
NKT: Natural killer T
NSCLC: Non-small-cell lung cancer
OS: Overall survival
PS: Performance Status
RFS: Recurrence-free survival
α-GalCer: α-galactosylceramide
